# Life beyond antibodies: quality of life after desensitization in kidney transplantation

**DOI:** 10.1093/ckj/sfae411

**Published:** 2025-01-27

**Authors:** Patrice Zoehinga, Thomas Jouve, Eloi Chevallier, Paolo Malvezzi, Lionel Rostaing, Johan Noble

**Affiliations:** Nephrology, Hemodialysis, Apheresis and Kidney Transplantation Department, University Hospital Grenoble, Grenoble, France; Nephrology, Hemodialysis, Apheresis and Kidney Transplantation Department, University Hospital Grenoble, Grenoble, France; Univ. Grenoble Alpes, CNRS, Inserm, U 1209 CNRS UMR 5309, Team Epigenetis Immunity, Metabolism, Cell Signaling and Cancer, Institute for Advanced Biosciences, Grenoble, France; Nephrology, Hemodialysis, Apheresis and Kidney Transplantation Department, University Hospital Grenoble, Grenoble, France; Nephrology, Hemodialysis, Apheresis and Kidney Transplantation Department, University Hospital Grenoble, Grenoble, France; Nephrology, Hemodialysis, Apheresis and Kidney Transplantation Department, University Hospital Grenoble, Grenoble, France; Nephrology, Hemodialysis, Apheresis and Kidney Transplantation Department, University Hospital Grenoble, Grenoble, France; Univ. Grenoble Alpes, CNRS, Inserm, U 1209 CNRS UMR 5309, Team Epigenetis Immunity, Metabolism, Cell Signaling and Cancer, Institute for Advanced Biosciences, Grenoble, France

To the Editor,

About 35% of kidney transplant candidates have pre-existing anti-human leukocyte antigen (HLA) and about 11% are highly sensitized, that is have a calculated panel reactive antigen (cPRA) >80% [1]. For highly sensitized patients with a PRA >99%, the access to kidney transplantation is very low [2]. The options for those patients are either waiting for a compatible donor, inclusion in an acceptable mismatch or paired donation program, or desensitization [3, 4, 5].

Different desensitization protocols have been studied. The most frequently used therapies to remove anti-HLA antibodies before kidney transplantation are anti-CD20 (rituximab), intravenous immunoglobulins, plasmapheresis (plasma exchange, double-filtration plasmapheresis or immunoadsorption) and imlifidase [6, 7]. However, other strategies have been tried or are under investigation such as anti-interleukin (IL)-6 (clazakizumab), anti-IL-6 receptor (tocilizumab), anti-CD38 and CAR-T cells [8, 9].

If transplantation improved recipient quality of life (QoL) as compared to dialysis, the benefit of desensitization, especially in the context of deceased donation, remains a topic of debate [10]. If those strategies allow an increase in access to kidney transplantation for highly sensitized patients, the rate of antibody-mediated rejection remains high (15–50% depending on the therapy and protocol biopsies) [3]. The patient mortality and death-censored graft survival have been reported to be comparable to non-desensitized highly sensitized living-donor kidney transplanted patients [11, 12, 13]. The survival benefit over patients who remain on the waiting list still needs to be demonstrated in deceased donors [14].

Desensitization, from clinical experience, can be challenging for the patient during the pre-transplantation period. Therefore, an unmet medical question remains if desensitization impacts QoL as compared to non-desensitized highly sensitized patients after kidney transplantation.

We conducted the first QoL study in desensitized patients at the Grenoble-Alpes hospital. The Medical Outcome Study Short Form-36 (MOS-SF36) was used as a general health scale and the ReTransQol (RTQ) as a kidney transplant recipient specific scale [15, 16].

Those two self-administrated questionnaire were assessed in highly sensitized patients (PRA ≥ 85%) who had received a kidney transplant after at least 3 months, with or without prior desensitization with anti-CD20 and plasmapheresis. The questionnaires were sent and returned electronically or by post between February and April 2022. The MOS-SF36 includes 36 items that evaluate eight dimensions (limitations in physical activities due to health problems, limitations in social activities due to physical or emotional issues, limitations in usual activities due to physical health problems, body pain, general mental health (psychological distress and well-being), limitations in usual activities due to emotional problems, vitality (energy and fatigue), and general health perceptions). The RTQ consists of 32 items that assess five dimensions (fear of losing the graft, physical health, mental health, medical care, and treatment). The scores for the different dimensions range from 0 to 100, with a higher score indicating a better QoL for the assessed dimension. Additionally, the patients’ demographic, clinical, and therapeutic characteristics were obtained from their electronic files. Numerical data are presented in this article as mean ± standard deviation and categorical values as number (percentage). For the patients’ characteristics, we used, after normality testing, the Kruskal–Wallis test to compare numerical values and chi-squared test for categorical data. R software was used to perform the statistical analysis and produce the figure. All medical data were collected from Grenoble database [CNIL (French National committee for data protection) approval number 1987785v0].

**Figure 1:**
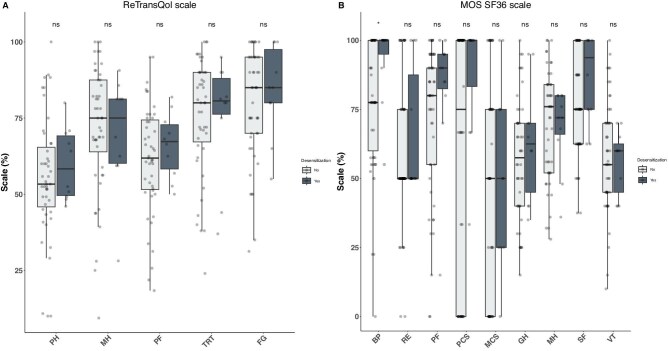
Quality of life scales between desensitized and non-desensitized highly sensitized kidney transplanted recipients. Panel A refers to the ReTransQol scale and panel B refers to the MOS-SF36 scale. PH, Physical health; MH, mental health; MC, medical care and satisfaction; TRT, treatment; FG, fear of losing the graft; PF, physical functioning; BP, bodily pain; GH, general health; VT, vitality; SF, social functioning; RE, role emotional; PCS, physical component score; MCS, mental component score

Sixty highly sensitized kidney transplant recipients were included. Among them, 11 (18.3%) were desensitized. Baseline characteristics of the desensitized and non-desensitized patients are reported in Table 1. Desensitized versus non-desensitized recipients received more frequently a kidney from a living donor (40% versus 2.2%, *p *< 0.001, respectively) and the time on waiting list was longer in desensitized patients that received a kidney from a deceased donor (6.3 ± 2.0 versus 3.2 ± 2.4 years, *p *= 0.006). The patients were similar regarding the other characteristics. There was no statistical difference between desensitized and non-desensitized kidney transplant recipients for the RTQ QoL scale (Fig. 1A). Regarding the MOS-SF36 scale, desensitized patients reported a significantly higher bodily pain score compared to non-desensitized patients (92.9% versus 80%, *p *= 0.019). There was no difference in the other dimensions of the MOS-SF36 scale (Fig. 1B). Of note, the questionnaire was given significantly sooner post-transplantation in desensitized patients: 25.9 months [31–64] compared to 87 months [38–120] in non-desensitized patients, *p *= 0.007.

**Table 1:** Baseline characterstics of the study population.

	Highly sensitized non-desensitized kidney transplant recipients (*N* = 49)	Highly sensitized desensitized kidney transplant recipients (*N* = 11)	*P*-value
Age—years	61 ± 12	53 ± 18	0.20
Gender, M/F ratio	0.44	0.57	0.71
Nephropathy			0.30
IgA	5 (10.2%)	1 (9.1%)	
Membranous *N*	4 (8.2%)	1 (9.1%)	
Urologic	9 (18.4%)	0	
Tubular necrosis	0	2 (18.2%)	
Deceased donor	43 (95.6%)	6 (60%)	<0.001
cPRA	94 [90–98]	97 [94–99]	0.15
Waiting time before Tx (years)			
All patients	3.4 ± 2.4	3.9 ± 3.1	0.886
Deceased donors	3.2 ± 2.4	6.3 ± 2.0	0.006
Diabetes	6 (13.3%)	1 (9.1%)	0.70
Hypertension	32 (71.1%)	7 (63.6%)	0.74
Chronic heart disease	1 (2.2%)	0	0.62
Chronic obstructive pulmonary disease	1 (2.2%)	0	0.77
Smoking	4 (9.1%)	4 (36.4%)	0.17
Time between Tx and questionnaire (months)	25.9 [31–64]	87 [38–120]	0.007
Time of rejection post Tx	1.6 [0.6–2]	2.7 [0.9–3.2]	0.699

cPRA: calculated Panel Reactive Antigen, Tx: Transplantation.

This study is the first QoL study in desensitization kidney recipients (plasmapheresis and anti-CD20) as compared to non-desensitized kidney recipients. We use a health scale generally well-recognized in the literature (MOS-SF36) and a scale developed specifically for transplanted patients (RTQ). We showed that the QoL is not impaired by desensitization and is relatively similar to non-desensitized patients, except for the bodily pain dimension. There are some limitations in this study, for example the limited number of patients included and the absence of inclusion of patients remaining on the waiting list, a different delay of questionnaire assessment between the two groups. In patients who received a kidney from a deceased donor, the time on the waiting list was longer in desensitized patients, which may participate to higher QoL scores.

We showed that desensitization does not impact significantly the QoL of recipients after kidney transplantation compared to non-desensitized patients and allows a faster access to transplantation, especially in patients with a very high PRA > 99%.
